# Postoperative cognitive dysfunction after inhalational anesthesia in elderly patients undergoing major surgery: the influence of anesthetic technique, cerebral injury and systemic inflammation

**DOI:** 10.1186/s12871-015-0130-9

**Published:** 2015-10-23

**Authors:** Yong Qiao, Hao Feng, Tao Zhao, Heng Yan, He Zhang, Xin Zhao

**Affiliations:** 1 Department of Anesthesiology, the Second Affiliated Hospital of Shandong University, Ji’nan, Shandong 250033 China; 2 Department of Anesthesiology, Rizhao People’s Hospital, Ri’zhao, 276000 Shandong China

**Keywords:** POCD, Sevoflurane inhalation anesthesia, TNF-α, IL-6, S-100β protein

## Abstract

**Background:**

Elderly patients are reportedly at higher risk of postoperative cognitive dysfunction (POCD) after inhalational anesthesia with sevoflurane. We hypothesized that the incidence of POCD would be higher in elderly patients undergoing major surgery under inhalational rather than intravenous anesthesia. We also measured plasma S-100β protein concentration as a biomarker of central nervous system injury, and plasma interleukin (IL)-6 and tumor necrosis factor (TNF)-α concentrations to judge the contribution of systemic inflammation to POCD.

**Methods:**

Ninety patients aged 65–75 years scheduled for resection of an esophageal carcinoma were randomly assigned to one of three groups (*n* = 30) as follows: a group receiving sevoflurane anesthesia (Group S); a group receiving preoperative methylprednisolone before sevoflurane anesthesia (Group S + MP); and a control group maintained with intravenous propofol (Group C). The mini-mental state examination (MMSE) and Montreal cognitive assessment (MoCA) were used to measure patients’ cognitive function the day before surgery, and on the first, third and seventh postoperative days. The plasma concentrations of TNF-α, IL-6 and S-100β protein were measured 10 min before anesthesia, and on the first, third and seventh postoperative days.

**Results:**

There were no significant differences in the demographic or clinical characteristics, or perioperative hemodynamic status, of the three groups. The MMSE and MoCA scores were significantly lower in Group S than in the propofol control (Group C) and Group S + MP on the first, third and seventh postoperative days (*P* <0.05). Throughout the first postoperative week the plasma concentrations of TNF-α, IL-6, and S-100β protein were significantly elevated in Group S compared with Group C (*P* <0.05), but were significantly lower in Group S + MP than Group S (*P* <0.05).

**Conclusions:**

The incidence of POCD was higher in elderly patients undergoing major surgery under inhalational anesthesia with sevoflurane than those maintained on intravenous propofol, and lower in elderly patients pro-treating with methylprednisolone. Furthermore, we found elevated plasma concentrations of S-100β protein, TNF-α and IL-6 in those receiving sevoflurane anesthesia.

**Trial registration:**

ChiCTR-IOR-15007007 (02-09-2015).

## Background

Postoperative cognitive dysfunction (POCD) is characterized by progressive hypomnesia, personality change or deterioration in cognitive function after surgery [1]. The incidence of POCD is rising steadily in patients undergoing general anesthesia [2, 3]. Age is the main risk factor for POCD, and although it is well recognized that the incidence of POCD is higher in the elderly [4], particularly those undergoing sevoflurane anesthesia [5], the mechanism is not fully understood.

There have been several reports that central nervous system inflammation provoked by anesthesia and surgery plays an important role in the pathogenesis of POCD [6]. Anesthesia and surgery have been shown to increase the brain concentration of interleukin (IL)-6, leading to neuronal apoptosis [7], and to provoke the release of the pro-inflammatory cytokine tumor necrosis factor (TNF)-α [8]. S-100β protein is an acidic calcium-binding protein found in the central nervous system, and when detected in the systemic circulation is considered a biomarker of acute brain injury [8, 9].

We undertook a randomized clinical trial to establish whether TNF-α, IL-6 or S-100β might play a role in the pathogenesis of POCD in elderly patients undergoing resection of an esophageal carcinoma under sevoflurane anesthesia with or without preoperative methylprednisolone, or under total intravenous anesthesia with propofol. Identifying a relationship between the serum concentrations of these pro-inflammatory cytokines and markers of cerebral injury would be an important step towards establishing strategies for the prevention of POCD.

## Methods

The study design was a prospective, double blind, randomized, controlled trial. It was conducted at the Second Affiliated Hospital of Shandong University, after approval had been obtained from the ethics committee of the Second Affiliated Hospital of Shandong University (IRB protocol number: SDUEY20121118). Informed consent was obtained from each participant.

### Patients

Ninety patients aged between 65 and 75 years undergoing esophageal carcinoma resection between January 2013 and December 2014 were enrolled. Patients were randomly assigned to one of three groups (*n* = 30) as follows: a group receiving sevoflurane anesthesia (Group S); a group receiving sevoflurane anesthesia after preoperative treatment with methylprednisolone (Group S + MP); and a control group of patients receiving intravenous propofol anesthesia (Group C).

The inclusion criteria were: American Society of Anesthesiologists (ASA) physical status I, II or III; a sufficient level of education to be capable of completing neuropsychological tests; a pre-operative mini mental state examination (MMSE) score ≥23; no evidence of cardiovascular, respiratory or central nervous system disease; normal renal and hepatic function; no serious hearing or visual impairment; absence of a history of benzodiazepine or antidepressant use, alcohol or cigarette misuse or drug dependence; and no contraindication to propofol or inhalational anesthesia.

### Patient management

Anesthesia was managed according to a standardized protocol that was identical for each group. Patients had fasted for 8 h and abstained from water for 4 h preoperatively. Arterial blood pressure, heart rate (HR), the electrocardiogram, peripheral blood oxygen saturation, bispectral index (BIS), end tidal CO_2_ (P_ET_CO_2_) and internal jugular central venous pressure were monitored in the operating room and recorded every 5 min.

Following 3 min of pre-oxygenation (with 100 % O_2_), anesthesia was induced by means of a single slow intravenous injection of midazolam (2–3 mg), etomidate (0.3 mg/kg) and an infusion of sufentanil (0.4 μg/kg). Tracheal intubation was facilitated with cisatracurium besylate (0.3 mg/kg). A left-sided double-lumen Robertshaw endobronchial tube (diameter Fr 35–39) was positioned using a laryngoscope.

Mechanical ventilation was initiated with a tidal volume of 7 ml/kg, a respiratory rate of 12 /min, a positive end expiratory pressure of 5 cmH_2_O and inspiratory–expiratory ratio of 1:2 to maintain P_ET_CO_2_ in the range 35–40 mmHg. Oxygen flow rate was set at 2 l/min.

Anesthesia was maintained in Group C by propofol administered by target controlled infusion (effect site concentration 4 ug/ml) and 1 minimum alveolar concentration (MAC) sevoflurane in Groups S and group S + MP. Intravenous infusion of methylprednisolone (10 mg/kg) was administered 30 min before anesthesia to patients in Group S + MP. Maintenance of anesthesia was supplemented by an intravenous infusion of remifentanil (commenced at 0.15 μg/kg/min and titrated according to clinical need). The sevoflurane or propofol was titrated on the basis of hemodynamic parameters (HR, systolic arterial blood pressure), to maintain BIS in the range 50–60, and if somatic (swallowing, movement) or autonomic signs (flushing, sweating, salivating) were evident. A 5-mg bolus of cisatracurium besylate was administered every 30 min according to clinical need. Sevoflurane and propofol were discontinued at the start of suturing.

Intravenous crystalloids were administered (0.9 % NaCl solution supplemented by lactated Ringer’s solution at a ratio of 3:1) at an infusion rate of 0.5–1.0 ml/kg/min for 20–40 min followed by an infusion rate of 0.25 ml/kg min to maintain a positive daily fluid balance and adequate urine output.

Postoperative analgesia (PCA: sufentanil 2 ug/kg) was provided using a patient-controlled intravenous technique for the first 72 h, with the following settings: 2 ml/h background infusion; 0.5-ml bolus, and a 15-min lockout.

Patients’ demographic and basic clinical characteristics, fluid balance, blood loss, duration of surgery, duration of single lung ventilation, temperature, time taken for recovery of spontaneous breathing, eye opening on command and time taken for extubation were also recorded. We recorded HR, mean arterial pressure (MAP) and central venous pressure at five perioperative time points: on entering the operating room (T0); before tracheal intubation (T1); before surgery (T2); immediately after surgery (T3) and immediately after tracheal extubation (T4).

### Enzyme-linked immunosorbent assay (ELISA)

Blood specimens were collected from the patients 10 min before anesthesia (Ta), and on the first (Tb), third (Tc), and seventh (Td) postoperative days. Plasma was obtained by centrifugation at 4000 × g for 20 min at 4 °C and stored at–80 °C. The concentrations of IL-6, TNF-α and S100β protein were measured using an ELISA kit according to the manufacturer’s instructions.

### Assessment of perioperative cognitive function

The MMSE and Montreal Cognitive Assessment (MoCA) were used to assess cognitive function the day before surgery (Ts), and on the first (Tb), third (Tc) and seventh (Td) postoperative days. All data collectors had undergone standard training, and were blinded to the randomization status of the participants. The MMSE consists of tests of orientation (in time and place), memory (immediate and short-term), calculation, language (naming, repetition, listening and reading comprehension, writing), visual spatial awareness, concentration and attention, and is suitable for detecting more severe cognitive dysfunction. The MoCA tests visuospatial and executive function (alternate trail making test, copy the cube, clock drawing), language ability, attention and calculation, delayed recall and abstract thinking, and can detect milder cognitive dysfunction. Patients’ cognitive function was evaluated by MoCA if the MMSE score equaled or exceeded 20.

### Statistical analysis

Assays were performed in triplicate, and representative examples of at least one assay are shown. All data are expressed as mean ± standard deviation (SD). Statistical differences were assessed using one-way analysis of variance (ANOVA) with the least significant difference (LSD) *post hoc* tests. A P value <0.05 was considered statistically significant. All statistical analyses were performed using the SPSS 19.0 statistics program for windows (SPSS, IBM, USA).

## Results

### Participants’ demographic and clinical characteristics

**Fig. 1:**
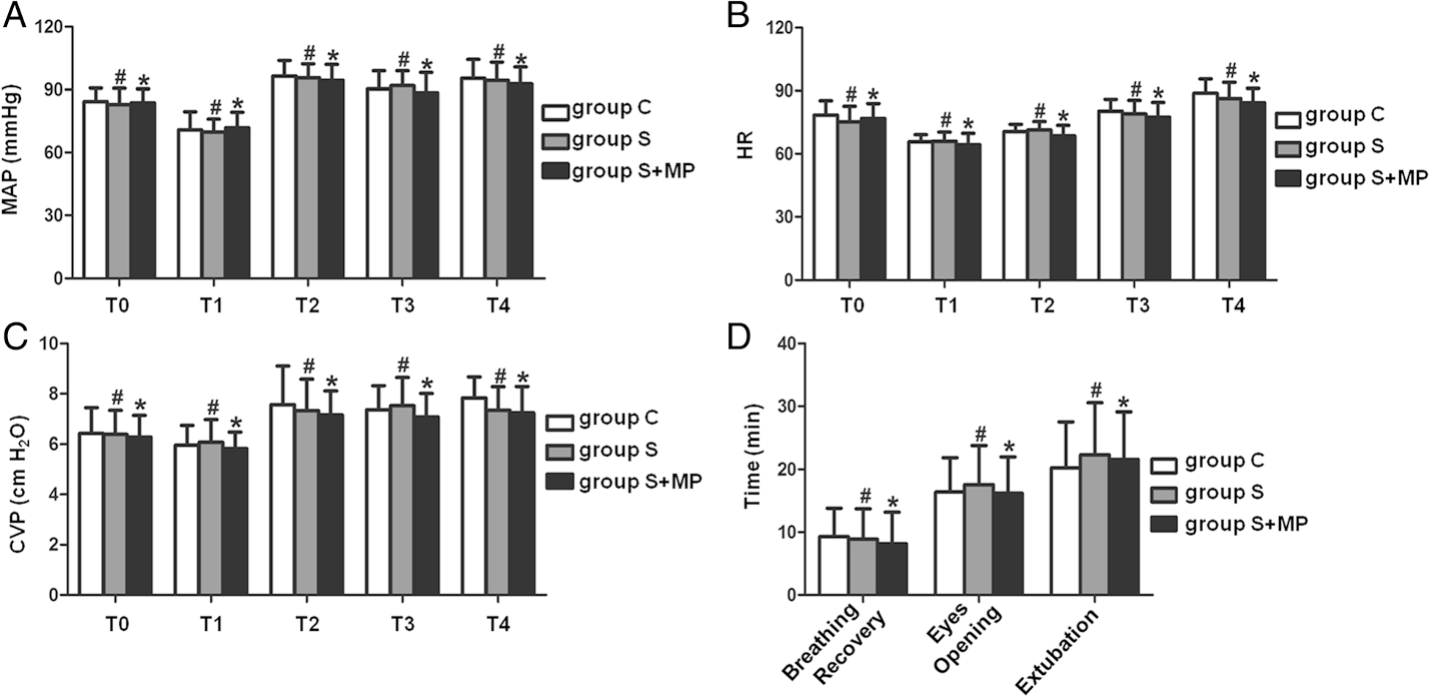
Comparison of HR (**a**), MAP (**b**), CVP (**c**), Time of breathing recovery, eyes opening, and extubation (**d**) in group C, group S, and group S + MP. HR, MAP, CVP were recorded at the time of entering the operating room (T0), the time before tracheal intubation (T1), the time before operation (T2), the time of the end of operation (T3), the time after tracheal extubation (T4) in group C, group S, and group S + MP. Compared with group C at corresponding time point, #*P* > 0.05 in group S, *P > 0.05 in group S + MP. Compared with the time of breathing recovery, eyes opening, and extubation in group C, #*P* > 0.05 in group S, *P > 0.05 in group S + MP. Data are representative of 30 independent experiments

There were no significant differences between the groups in terms of gender, age, body mass index (BMI) or preoperative MMSE scores (Table 1), nor were there significant differences in perioperative hemodynamic parameters (Fig. 1a, b, c), time to recovery of spontaneous breathing, emergence from anesthesia or time to extubation (Fig. 1d; Table 2).

**Table 1:** Clinical characteristics of patients

	Group C	Group S	Group S + MP	*P*-Value
Gender (n) Male/Female	21/9	22/8	21/9	0.832*
Age (yr)	68 ± 2	68 ± 3	69 ± 3	0.705
Body Mass Index (BMI)	24.41 ± 1.52	23.65 ± 1.14	24.24 ± 3.19	0.831
Preoperative MMSE Scores	28.62 ± 1.14	27.83 ± 1.79	27.45 ± 1.14	0.408
Duration of Surgery (min)	130 ± 5	128 ± 8	130 ± 5	0.786

Data are given as mean ± SD. *Fisher exact test

**Table 2:** Emergence from anaesthesia

	Group C	Group S	Group S + MP	*P*-Value
Breathing recovery	9.2 ± 4.5	8.9 ± 4.2	8.1 ± 5.0	0.918
Eyes opening	16.4 ± 5.5	17.6 ± 6.2	16.2 ± 5.7	0.912
Extubation	20.2 ± 7.2	22.2 ± 8.1	21.6 ± 7.5	0.914

All times are given as mean ± SD

### Cognitive function assessment and plasma S-100β concentration

**Fig. 2:**
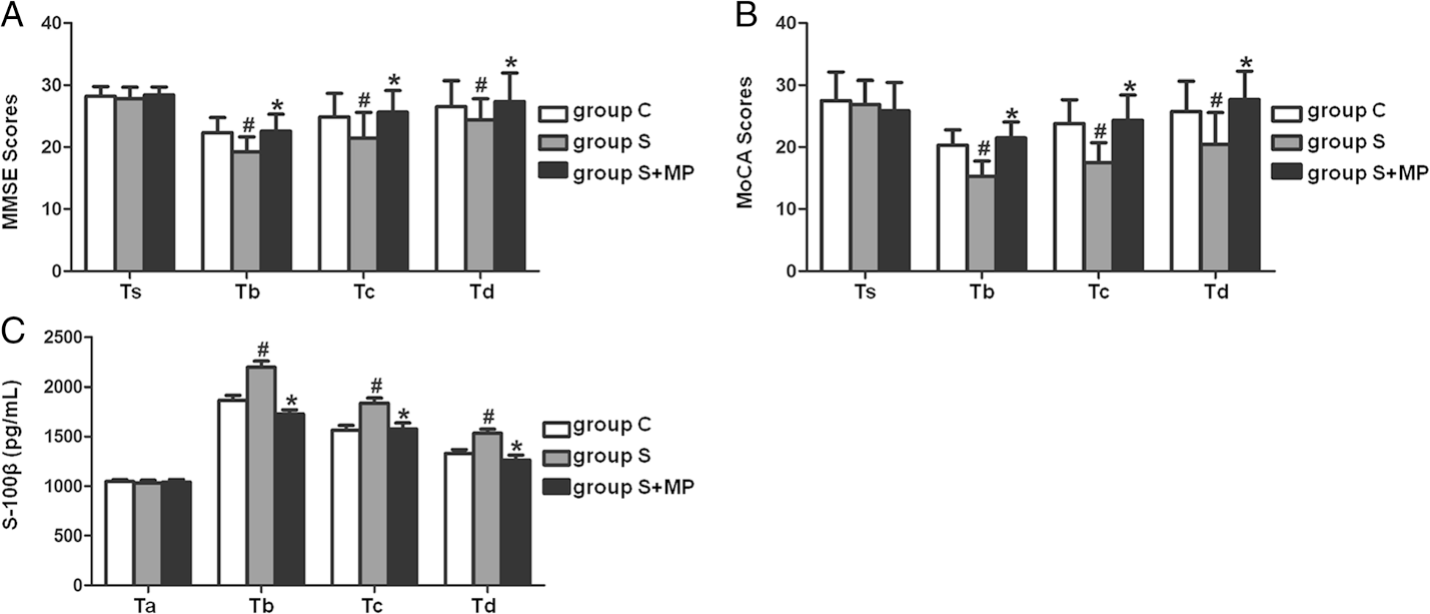
Comparison of MMSE and MoCA scores, S-100β protein level in group C, group S, and group S + MP. MMSE and MoCA scores were used to assess the cognitive function at the day before operation (Ts), the first (Tb), third (Tc), and seventh (Td) day after operation, and S-100β protein level was used to reflect the level of brain injury at 10 min before anesthesia (Ta), the first (Tb), third (Tc), and seventh (Td) day after operation in different groups. **a** and **b**: MMSE and MoCA scores in different groups. Compared with Group C at Tb, Tc, and Td, #*P* <0.05 in group S; Compared with Group S at Tb, Tc, and Td, *P <0.05 in group S + MP. **c**: S-100β protein level in different groups. Compared with Group C at Tb, Tc, and Td, #*P* <0.05 in group S; Compared with Group S at Tb, Tc, and Td, *P <0.05 in group S + MP. Data are representative of 30 independent experiments

The MMSE and MoCA scores on the first, third and seventh postoperative days were significantly lower in Group S than controls (*P* <0.05; Fig. 2a, b; Tables 3 and 4). Furthermore, the MMSE and MoCA scores on the first, third and seventh postoperative days were significantly higher in Group S + MP than Group S (*P* <0.05; Fig. 2a, b; Tables 3 and 4). The concentration of plasma S-100β protein was significantly higher in Group S than Group C and Group S + MP at all postoperative time points (*P* <0.05; Fig. 2c; Table 5).

**Table 3:** Comparison of the levels of MMSE in group C, group S, and group S + MP

	Group C	Group S	Group S + MP	*P1*-*Value*	*P2*-*Value*
Ts	28.16 ± 1.60	27.83 ± 1.94	28.33 ± 1.21	0.725	0.599
Tb	22.50 ± 2.26	19.17 ± 2.14	22.43 ± 2.43	0.023	0.023
Tc	24.67 ± 3.67	19.33 ± 3.83	25.67 ± 2.34	0.015	0.005
Td	26.67 ± 3.67	20.33 ± 3.39	27.33 ± 4.37	0.012	0.006

Data are given as mean ± SD. *P1*-*Value*, group S *versus* group C (LSD). *P2*-*Value*, group S + MP *versus* group S (LSD)

**Table 4:** Comparison of the levels of MoCA in group C, group S, and group S + MP

	Group C	Group S	Group S + MP	*P1*-*Value*	*P2*-*Value*
Ts	27.55 ± 4.58	26.90 ± 3.83	25.95 ± 2.61	0.769	0.684
Tb	20.33 ± 2.34	15.33 ± 2.58	21.48 ± 2.43	0.003	0.001
Tc	23.83 ± 3.87	17.83 ± 2.99	24.33 ± 3.33	0.008	0.005
Td	25.33 ± 4.89	18.01 ± 4.15	27.67 ± 4.41	0.013	0.002

Data are given as mean ± SD. *P1*-*Value*, group S *versus* group C (LSD). *P2*-*Value*, group S + MP *versus* group S (LSD)

**Table 5:** Comparison of the levels of S100 β in group C, group S, and group S + MP

	Group C	Group S	Group S + MP	*P1*-*Value*	*P2*-*Value*
Ta	1043.42 ± 20.73	1028.45 ± 28.73	1038.61 ± 26.65	0.327	0.503
Tb	1864.93 ± 50.51	2194.28 ± 63.72	1728.87 ± 40.31	0.000	0.000
Tc	1562.37 ± 48.08	1836.55 ± 52.37	1576.73 ± 58.59	0.000	0.000
Td	1328.83 ± 38.22	1531.60 ± 42.83	1261.50 ± 50.15	0.000	0.000

Data are given as mean ± SD. *P1*-*Value*, group S *versus* group C (LSD). *P2*-*Value*, group S + MP *versus* group S (LSD)

### Plasma IL-6 and TNF-α concentrations

**Fig. 3:**
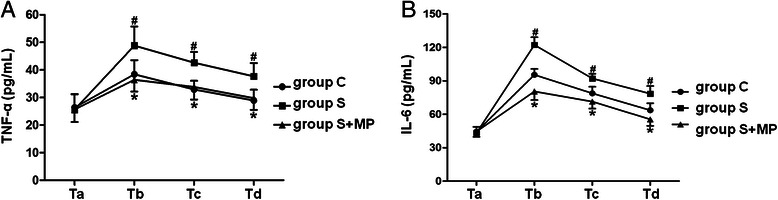
Comparison of the levels of TNF-α, IL-6 in group C, group S, and group S + MP. The levels of TNF-α, IL-6 were used to reflect the systemic inflammatory response at 10 min before anesthesia (Ta), the first (Tb), third (Tc), and seventh (Td) day after operation in group C, group S, and group S + MP. **a**: the level of TNF-α in different groups. Compared with Group C at Tb, Tc, and Td, #*P* <0.05 in group S; Compared with Group S at Tb, Tc, and Td, *P <0.05 in group S + MP. **b**: the level of IL-6 in different groups. Compared with Group C at Tb, Tc, and Td, #*P* <0.05 in group S; Compared with Group S at Tb, Tc, and Td, *P <0.05 in group S + MP. Data are representative of 30 independent experiments

The plasma concentrations of IL-6 and TNF-α were significantly higher in Group S on the first, third and seventh postoperative days than controls (*P* <0.05; Fig. 3a, b; Tables 6 and 7), and were significantly lower in group S than those in Group S + MP (*P* <0.05; Fig. 3a, b; Tables 6 and 7) at each time point.

**Table 6:** Comparison of the levels of IL-6 in group C, group S, and group S + MP

	Group C	Group S	Group S + MP	*P1*-*Value*	*P2*-*Value*
Ta	44.33 ± 4.41	43.25 ± 5.40	44.52 ± 4.79	0.707	0.664
Tb	95.40 ± 5.31	122.23 ± 6.85	80.48 ± 7.71	0.000	0.000
Tc	78.75 ± 5.98	92.11 ± 4.26	71.32 ± 6.18	0.001	0.000
Td	63.58 ± 6.33	78.43 ± 6.95	55.42 ± 5.82	0.001	0.000

Data are given as mean ± SD. *P1*-*Value*, group S *versus* group C (LSD). *P2*-*Value*, group S + MP *versus* group S (LSD)

**Table 7:** Comparison of the levels of TNF-α in group C, group S, and group S + MP

	Group C	Group S	Group S + MP	*P1*-*Value*	*P2*-*Value*
Ta	26.37 ± 4.73	25.65 ± 5.62	25.68 ± 4.56	0.807	0.991
Tb	38.41 ± 5.13	48.82 ± 6.89	36.43 ± 4.27	0.005	0.002
Tc	32.92 ± 3.16	42.63 ± 3.87	33.83 ± 4.54	0.001	0.001
Td	28.92 ± 3.90	37.65 ± 4.84	29.80 ± 4.28	0.003	0.007

Data are given as mean ± SD. *P1*-*Value*, group S *versus* group C (LSD). *P2*-*Value*, group S + MP *versus* group S (LSD)

## Discussion

We found that the incidence of POCD in elderly patients undergoing major surgery was higher in those receiving sevoflurane general anesthesia than those receiving total intravenous anesthesia with propofol. We also found that methylprednisolone afforded a degree of neuroprotection in those receiving sevoflurane anesthesia, and that POCD was associated with elevated postoperative plasma concentrations of TNF-α, IL-6 and S100β protein.

Postoperative cognitive dysfunction may be manifest as impairment of working memory, long-term memory, information processing, attention or cognitive flexibility [10], adversely affecting quality of life, social independence and mortality [11]. It may persist for weeks or months, and may not resolve at all in a small proportion of those affected [12].

Inhalational anesthetic drugs are reported to promote neuronal apoptosis in animal models, leading to a decline in learning ability and memory after anesthesia [13–15]. Uemura et al. documented behavioral abnormalities in adult rats that had been exposed to halothane *in utero* [16]. Exposure to a low concentration of halothane or N_2_O reportedly causes visual impairment, and reduces instantaneous memory, and athletic and cognitive abilities [17]. Post-exposure cognitive function is also reportedly superior in patients anesthetized with intravenous propofol than those treated with sevoflurane [18].

Our findings are consistent with this body of research. We documented a substantial reduction in MMSE and MoCA scores in the first postoperative week in those exposed to sevoflurane rather than propofol. This suggests that sevoflurane increases the risk of POCD in elderly patients undergoing major surgery.

The presence of S100β protein in the systemic circulation is an indicator of central nervous system injury. With increasing age, ongoing demyelination leads to an increase in the plasma concentration and half-life of S-100β protein [19]. The physiological role of S100β protein, which is produced by astrocytes, is to enhance interaction between neurons and glial cells [20]. An increased concentration of S100β protein is indicative of a severe brain injury [21]. We found that the plasma concentration of S100β protein was significantly more elevated throughout the first postoperative week in those anesthetized with sevoflurane compared with those who received propofol. This suggests that plasma S100β protein concentration could be used as a biomarker of POCD.

In the elderly, POCD may persist for weeks or months after inhalational anesthesia and a small minority of patients is affected for longer; however, the pathophysiologic mechanisms are still not fully understood. Propofol is reported to inhibit the activation and release of inflammatory factors such as IL-6 and TNF-α by astrocytes in the central nervous system [22, 23]. This implicates IL-6 and TNF-α in the pathogenesis of POCD caused by sevoflurane.

Interleukin-6 is an important regulator of synapse formation; a high local concentration of IL-6 is reported to inhibit synaptic function. Hippocampal neurogenesis in the dentate gyrus is reported to be decreased by 63 % in adult transgenic rats that overexpress IL-6 from their astrocytes [24]. Administration of a neutralizing antibody for IL-6 also significantly improves long-term potentiation (LTP) and spatial memory in rats. The influence of IL-6 on the generation of LTP, and its inhibitory effects on learning and memory, suggest it is likely to also play a role in POCD [25].

Tumor necrosis factor-α is a cell signaling protein involved in innate and specific immunity, and promotes the inflammatory response [26, 27]. Monocytes and macrophages activated by external stimuli release TNF-α, which in turn promotes the release of other inflammatory mediators and pro-inflammatory cytokines such as IL-6, thus starting the inflammatory cascade reaction. Isoflurane anesthesia is reported to increase the incidence of POCD in diabetic rats by a TNF-α–dependent mechanism [28].

We found that the plasma concentrations of IL-6 and TNF-α were significantly higher throughout the first postoperative week in those exposed to sevoflurane than propofol. Furthermore, the plasma concentrations of IL-6 and TNF-α were significantly lower in those undergoing sevoflurane anesthesia if they had been administered methylprednisolone preoperatively. This suggests that the pro-inflammatory cytokines IL-6 and TNF-α may be part of the mechanism underlying the pathophysiology of POCD in elderly patients undergoing major surgery.

Methylprednisolone (10 ug/mL) could suppress lymphocyte blastoid transformation, immunoglobulin production, and inflammatory action [29]. This study showed that preoperative methylprednisolone administration suppressed postoperative increases in the plasma levels of IL-6 and TNF-α. Methylprednisolone may be an effective means of reducing the incidence of POCD in elderly patients caused by sevoflurane inhalation anesthesia by suppressing the level of IL-6 and TNF-α.

We found a higher incidence of POCD in patients undergoing major surgery under sevoflurane anesthesia, and an association with increased plasma concentrations of IL-6 and TNF-α. Preoperative treatment with methylprednisolone may afford a degree of protection against POCD, raising the possibility that it could be used prophylactically in elderly patients undergoing major surgery.

## Conclusions

We found that the incidence of POCD was higher in patients undergoing sevoflurane anesthesia for major surgery than those receiving an intravenous propofol regime, and lower in elderly patients pro-treating with methylprednisolone. The MMSE and MoCA were useful tools for the detection of POCD, and plasma S-100β protein concentration may be a useful biomarker of disease. Furthermore, POCD was associated with elevated plasma concentrations of IL-6 and TNF-α.

## Abbreviations

POCD: Postoperative cognitive
MMSE: Mini-mental state examination
MoCA: Montreal cognitive assessment
TNF-α: Tumor necrosis factor-α
IL-6: Interleukin-6
BP: Blood pressure
HR: Heart rate
CVP: Central venous pressure
BIS: Bispectral index
ECG: Electrocardiography
ELISA: Enzyme-linked immunosorbent assay
ANOVA: One-way analysis of variance
